# GAS8 and GAS8-AS1 expression in gastric cancer 

**Published:** 2019

**Authors:** Farbod Esfandi, Fatemeh Mohammad Rezaei, Mohammad Taheri, Maryam Naby Gol, Vahid Kholghi Oskooei, Amir Namvar, Soudeh Ghafouri-Fard

**Affiliations:** 1 *Department of Medical Genetics, Shahid Beheshti University of Medical Sciences, Tehran, Iran*; 2 *Department of Medical Genetics, Faculty of Medicine, Tabriz University of Medical Sciences, Tabriz, Iran*; 3 *Urogenital Stem Cell Research Center, Shahid Beheshti University of Medical Sciences, Tehran, Iran*; 4 *Student Research Committee, Qom University of Medical Sciences, Qom, Iran *

**Keywords:** GAS8, RNA, Long noncoding, Gastric cancer

## Abstract

**Aim:**

To evaluate the expression of the growth arrest-specific 8 (GAS8) and its antisense (GAS8-AS1) in gastric cancer.

**Background:**

GAS8 exists in a genomic region that is recurrently deleted in breast and prostate cancer. This gene contains a long non-coding RNA, namely GAS8-AS1 whose roles in the regulation of GAS8 has been reported in hepatocytes. GAS8-AS1 has also been regarded as a putative tumor suppressor gene in papillary thyroid cancer and hepatocellular carcinoma.

**Methods:**

In the present study, we evaluated expression levels of GAS8 and GAS8-AS1 in 30 gastric cancer tissues and their corresponding adjacent non-cancerous tissues (ANCTs).

**Results:**

GAS8 was significantly down-regulated in tumor tissues compared to ANCTs (Expression ratio=0.29, p<0.001). Although the expression of GAS8-AS1 was higher in tumor tissues compared to ANCTs (Expression ratio=2.15), it did not reach the level of significance (p=0.12). GAS8 expression was associated with the site of the primary tumor (p=0.01). GAS8-AS1 expression was significantly higher in tumors with lymphatic/ vascular invasion compared with those without lymphatic/ vascular invasion (p=0.03). Significant pairwise correlations were detected between expression levels of GAS8 and GAS8-AS1 in tumor tissues and ANCTs. Based on the results of the ROC curve, the diagnostic power of transcript levels of GAS8 in gastric tissues was estimated to be 76%.

**Conclusion:**

The current study underscores the roles of GAS8 and GAS8-AS1 in gastric carcinogenesis and warrants future functional studies to unravel the underlying mechanism of such contribution.

## Introduction

 The *growth arrest-specific 8* (*GAS8*) resides in a genomic region that is recurrently deleted in breast and prostate cancer. A long non-coding RNA (lncRNA), namely *C16orf3* (*GAS8-antisense 1* or *GAS8-AS1*) is located in the second intron of this gene and is transcribed in the opposite orientation (1). Although the function of its mouse homolog is associated with growth arrest, a previous study in breast cancer failed to find mutations in *GAS8* and *GAS8-AS1* in tumor DNA (1). GAS8 is a microtubule-binding protein that participated in the control of dynein function (2). Germline mutations in *GAS8* gene have been associated with primary ciliary dyskinesia-33 (CILD33; 616726) (3). Although the studies on the role of GAS8 in carcinogenesis process are scarce, several recent studies have focused on expression level and role of the antisense gene (*GAS8-AS1*). This lncRNA has been identified as the second most commonly mutated gene in papillary thyroid carcinoma (PTC) samples of Chinese patients (4). Functional studies in PTC cell lines have shown the role of *GAS8-AS1* in the suppression of cell proliferation and induction of autophagy via modulation of ATG5 expression (5).

Moreover, plasma levels of this lncRNA were lower in PTC patients compared with its levels in nodular goiters. Notably, decreased *GAS8-AS1* plasma concentration was associated with lymph node metastasis in these patients (6). The role of *GAS8* and *GAS8-AS1* in liver cancer has also been evaluated. *GAS8-AS1* has been shown to increase GAS8 expression by preserving the active chromatin configuration in the *GAS8* promoter. The consequent GAS8 over-expression has suppressed the malignant transformation of liver cells (7). Based on the proposed roles for *GAS8* and *GAS8-AS1* in the suppression of tumorigenesis and lack of data regarding their role in gastric carcinogenesis, in the current research, we assessed the expression of these genes in tissues obtained from gastric cancer patients. 

## Methods

**Patients**

A total of 60 gastric tissue specimens, including gastric tumors (n=30) and adjacent non-cancerous tissues (ANCTs) (n=30) from the same patients, were acquired for the current study. Tissues were excised from patients during surgery as a routine step in their treatment. Patients had no previous chemo/radiotherapy before tissue removal. A pathologist confirmed the diagnosis. The ethical committee approved the study protocol of Shahid Beheshti University of Medical Sciences. All patients have signed written informed consent forms. 

**Expression assay**

Expression studies were conducted on total RNA extracted from tissues using TRIzol™ Reagent (Invitrogen, Carlsbad, CA, USA). cDNA was synthesized from RNA using Applied Biosystems High-Capacity cDNA Reverse Transcription Kit. Expressions of *GAS8* and *GAS8-AS1* were quantified in the Rotor-Gene 6000 Real-Time PCR Machine using TaqMan® Universal PCR Master Mix (Applied Biosystems, Foster City, CA). *HPRT1* was used as normalizer. The nucleotide sequences of primers and probes and PCR product length are summarized in table 1.

**Statistical analysis**

Expression levels of *GAS8* and *GAS8-AS1* in gastric tumor tissues and ANCTs were compared using REST 2009 software. The significance of the difference in their expression between these two sets of samples was assessed using the Student’s paired t-test. The association between clinical characteristics and relative expression of genes was evaluated using the Chi-square test and the Mann-Whitney test. The correlation between transcript levels of *GAS8* and *GAS8-AS1* was measured using the regression model. P<0.05 was considered significant. The diagnostic power of transcript levels of these genes was assessed by depicting the receiver operating characteristic curve (ROC). 

## Results

**General demographic and clinical data**

The clinical and demographic data of study participants are summarized in table 2.

**Relative expressions of genes in tumor tissues vs. ANCTs**

**Figure 1:**
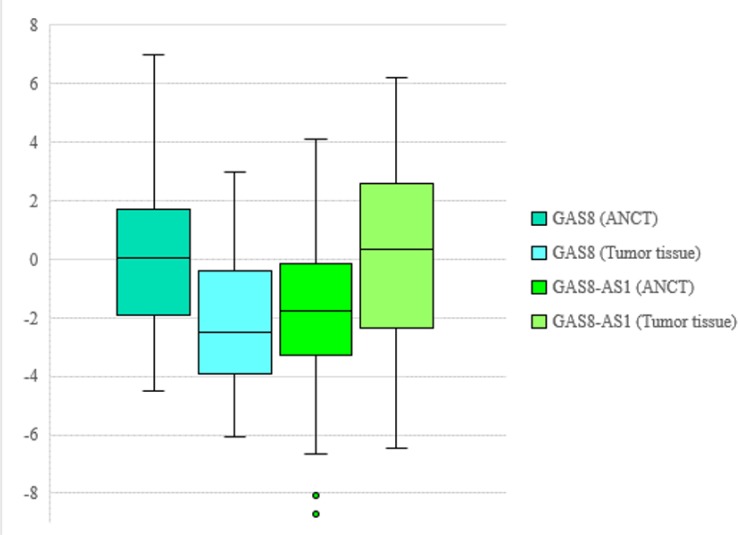
Relative expression of GAS8 and GAS8-AS1 in gastric cancer samples (n=30) and ANCTs (n=30) as designated by –delta CT values (CT HPRT1- CT target gene)

*GAS8* was significantly down-regulated in tumor tissues compared to ANCTs (Expression ratio=0.29, p<0.001). Although the expression of *GAS8-AS1* was higher in tumor tissues compared to ANCTs (Expression ratio=2.15), it did not reach the level of significance (p=0.12) (Figure 1). 

**Table 1:** The primers and probes sequences and PCR product length

Product length	Primer and probe length	Primer and probe sequence	Gene name
88	18	F: AGCCTAAGATGAGAGTTC	*HPRT1*
	21	R: CACAGAACTAGAACATTGATA	
	24	FAM -CATCTGGAGTCCTATTGACATCGC- TAMRA	
121	22	F: CTACAACGACATCACCCTCAAC	*GAS8*
	20	R: GTTCTGCCCAGACACCTCTG	
	24	FAM- TCTCCCTCTCCAGGTGGTCCTCCT -TAMRA	
144	20	F: CCCATAGCCTGCCCCGTAAG	*GAS8-AS1*
	20	R: CGTTGTCCCAGCATGTGAGC	
	24	FAM -CCCGTCTCCCTGTCCGCTTCCCAT-TAMRA	

**Table 2:** The clinical and demographic data of study participants

Variables		Values
Age (mean ± SD (range))		42.5±10.1(14-55)
Gender	Male	78.6%
	Female	21.4%
Site of the primary tumor	Cardia	41.4%
	Antrum	31%
	Body	27.6%
Histologic grade	2	37.5%
	3	58.3%
	4	4.2%
Lymphatic invasion	Yes	82.8%
	No	17.2%
Vascular invasion	Yes	82.8%
	No	17.2%
Peritoneal invasion	Yes	62.1%
	No	37.9%
TNM stage	I	3.4%
	II	31%
	III	44.8%
	IV	20.8%
Histological form	Intestinal	46.7%
	Diffuse	53.3%
*H. pylori *infection	Positive	50%
	Negative	50%
Smoking	Never Smoker	50%
	Current Smoker	13.6%
	Ex-Smoker	36.4%

**Association between genes expression and clinical features**

*GAS8* expression was associated with the site of the primary tumor (p=0.01). Other variables were not related to the expression of either *GAS8* or *GAS8-AS1* (table 3).

**Table 3:** The results of association analysis between relative expressions of *GAS8 *and *GAS8-AS1* in gastric cancer tissues compared to ANCTs and tumor features (Up/down regulation of genes was delineated according to the relative expression of each gene in tumor tissue compared to its paired ANCT).

	*GAS8 *up-regulation	*GAS8 *down-regulation	P value	*GAS8-AS1 *up-regulation	*GAS8-AS1 *down-regulation	P value
Age			0.64			0.64
> 50	6 (28.6%)	15 (71.4%)		15 (71.4%)	6 (28.6%)	
≤ 50	3 (42.9%)	4 (57.1%)		4 (57.1%)	3 (42.9%)	
Gender			0.64			0.14
Female	1 (16.7%)	5 (83.3%)		2 (33.3%)	4 (66.7%)	
Male	7 (31.8%)	15 (68.2%)		16 (72.7%)	6 (27.3%)	
Site of primary tumor			0.01			0.18
Cardia	1 (8.3%)	11 (91.7%)		6 (50%)	6 (50%)	
Antrum	6 (66.7%)	3 (33.3%)		8 (88.9%)	1 (11.1%)	
Body	2 (25%)	6 (75%)		5 (62.5%)	3 (37.5%)	
Histology grade			0.33			0.61
2	2 (22.2%)	7 (77.8%)		6 (66.7%)	3 (33.3%)	
3	5 (35.7%)	9 (64.3%)		9 (64.3%)	5 (35.7%)	
4	1 (100%)	0 (0%)		0 (0%)	1 (100%)	
Lymphatic invasion			1			0.63
Yes	8 (33.3%)	16 (66.7%)		15 (62.5% )	9 (37.5 %)	
No	1 (20%)	4 (80%)		4 (80%)	1 (20%)	
Vascular invasion			1			0.63
Yes	8 (33.3%)	16 (66.7%)		15 (62.5% )	9 (37.5 %)	
No	1 (20%)	4 (80%)		4 (80%)	1 (20%)	
Peritoneal invasion			1			0.69
Yes	6 (33.3%)	12 (66.7%)		11 (61.1%)	7 (38.9%)	
No	3 (27.3%)	8 (72.7%)		8 (72.7%)	3 (27.3%)	
Pathological T			1			0.09
T2b	1 (25%)	3 (75%)		2 (50%)	2 (50%)	
T3	5 (29.4%)	12 (70.6%)		9 (52.9%)	8 (47.1%)	
T4	2 (33.3%)	4 (66.7%)		6 (100%)	0 (0%)	
Pathological N			0.15			0.35
N0	2 (22.2%)	7 (77.8%)		6 (66.7%)	3 (33.3%)	
N1	2 (22.2%)	7 (77.8%)		5 (55.6%)	4 (44.4%)	
N2	5 (62.5%)	3 (37.5%)		7 (87.5%)	1 (12.5%)	
N3	0 (0%)	3 (100%)		1 (33.3%)	2 (66.7%)	
TNM Staging			0.91			0.45
I	0 (0%)	1 (100%)		1 (100%)	0 (0%)	
II	2 (22.2%)	7 (77.8%)		4 (44.4%)	5 (55.6%)	
III	5 (38.5%)	8 (61.5%)		9 (69.2%)	4 (30.8%)	
IV	2 (33.3%)	4 (66.7%)		5 (83.3%)	1 (16.7%)	
Histological form			0.69			0.51
Intestinal	5 (35.7%)	9 (64.3%)		8 (57.1%)	6 (42.9%)	
Diffuse	4 (25%)	12 (75%)		11 (68.7%)	5 (31.3%)	
*H pylori* Infection			1			0.7
Positive	4 (26.7%)	11 (73.3%)		10 (66.7%)	5 (33.3%)	
Negative	8 (33.3%)	10 (66.7%)		9 (60%)	6 (40%)	
Smoking			1			1
Non-Smoker	2 (18.2%)	9 (81.8%)		7 (63.6%)	4 (36.4%)	
Smoker	1 (33.3%)	2 (66.7%)		2 (66.7%)	1 (33.3%)	
Ex- Smoker	2 (25%)	6 (75%)		6 (75%)	2 (25%)	

Table 3. The results of association analysis between relative expressions of *GAS8 *and *GAS8-AS1* in gastric cancer tissues compared to ANCTs and tumor features (up/down regulation of genes was delineated according to the relative expression of each gene in tumor tissue compared to its paired ANCT).

We calculated relative values for expression of each gene in tumor tissues based on the following equation: Efficiency ^CT reference gene-Efficiency ^CT target gene. Subsequently, we assessed associations between these values and clinical variables using Mann–Whitney U test (table 4). *GAS8-AS1 *expression was significantly higher in tumors with lymphatic/ vascular invasion compared with those without lymphatic/ vascular invasion (p=0.03). No other significant associations were detected between expression levels of genes in tumor tissues and clinical data.

**Table 4:** Association between expression levels of genes in tumor tissues and clinical data (Mean (Standard deviation) values of Efficiency ^CT reference gene-Efficiency ^CT target gene are displayed)

	*GAS8*	P value	*GAS8-AS1*	P value
Age				
<50 years old vs. ≥50 years old	155.49 (546.31) vs. 10.86 (27.88)	0.11	90.5 (296.33) vs. 52.99 (87.34)	0.29
Lymphatic invasion				
Yes vs. No	138.86 (511.58) vs. 1.72 (2.66)	0.41	94.61 (287.11) vs. 0. 2 (0. 2)	0.03
Vascular invasion				
Yes vs. No	138.86 (511.58) vs. 1.72 (2.66)	0.41	94.61 (287.11) vs. 0. 2 (0. 2)	0.03
*H.pylori* Infection				
Positive vs. Negative	212.72 (643.44) vs. 24.91 (58.45)	0.25	138.81 (347.67) vs. 12.72 (26.75)	0.77
Tumor grade				
Grade 2 vs. 3 and 4	171.9 (628.01) vs. 80.7 (239.21)	0.72	58..47 (104.45) vs. 154.29 (443.77)	0.77

**Table 5:** Complete elements of ROC curve analysis (a: Youden index, b: Significance level P (Area=0.5), Estimate criterion: optimal cut-off point for gene expression)

Gene name	Estimate criterion	AUC	J^a^	Sensitivity	Specificity	P-value^b^
*GAS8*	> 2. 14	0.76	0.43	0.63	0.80	< 0.000

**Figure 2:**
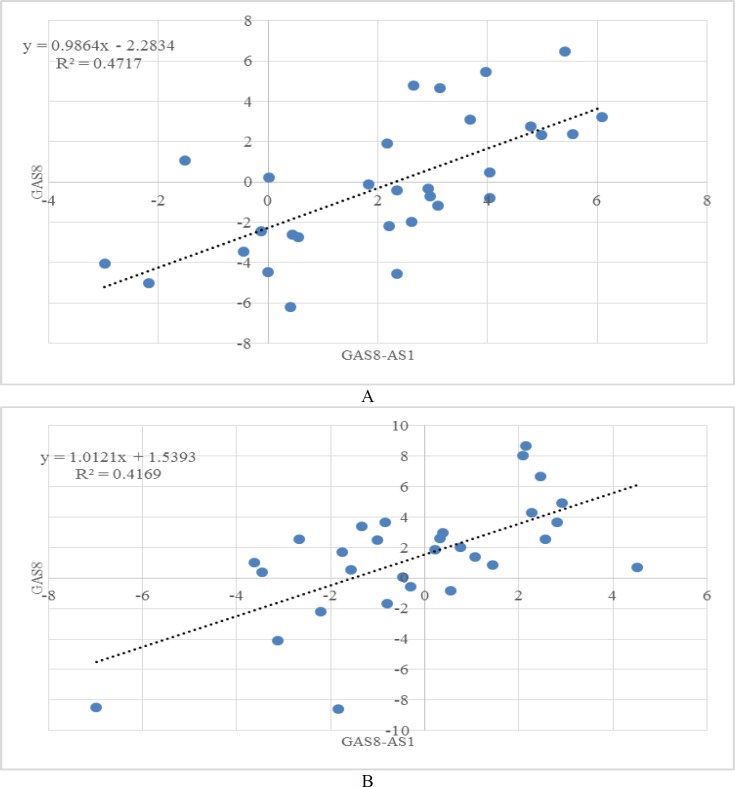
Correlations between expression of GAS8 and GAS8-AS1 in tumor tissues (A) and non-tumoral tissues (B).

**Correlations between expression levels of** ***GAS8*** **and** ***GAS8-AS1*** **in each set of samples**

Significant pairwise correlations were detected between expression levels of *GAS8* and *GAS8-AS1* in tumor tissues (Figure 2A) and ANCTs (Figure 2B).

**Receiver operating characteristic** **(ROC) curve analysis**

Based on the results of the ROC curve, the specificity and sensitivity values of *GAS8* transcript levels were 0.8, and 0.63, respectively (Table 5). The diagnostic power of transcript levels of this gene was estimated to be 76%.

## Discussion

In the present project, we evaluated expression levels of *GAS8* and *GAS8-AS1* in gastric tissue samples and reported down-regulation of *GAS8* in tumor tissues compared to ANCTs. *GAS8* has been suggested as a tumor suppressor gene in some kinds of human cancers (4, 7). However, the role of this gene in gastric cancer has not assessed yet. Previous studies have evaluated the role of other *GAS* genes in gastric cancer. For instance, Wang *et al*. have reported down-regulation of *GAS1* in gastric cancer patients, especially in patients with poor clinical outcomes. Functional studies have shown the role of this gene in the suppression of cell proliferation both *in vitro* and *in vivo* (8). On the other hand, GAS6 has been demonstrated to participate in a signaling pathway which promotes cellular survival and invasion of gastric cancer cells through the Akt pathway (9). 

We also detected an association between expression of *GAS8 *and the site of the primary tumor in a way that in nearly all of the cardia tumors, *GAS8* was down-regulated compared with the paired ANCT. Such finding further emphasizes the difference in pathological features of the cardia and non-cardia gastric tumors (10) and potentiates *GAS8* expression levels as a biomarker for assessment of malignancy status in the cardia region.

Despite the previously reported role of *GAS8-AS1* in the suppression of tumor growth in some malignancies (5), we could not detect any significant difference in its expression between tumor tissues and ANCTs. However, based on the relatively small sample size, our results are not conclusive. So, we suggest further study of its expression both in cancer cell lines and in clinical samples. This suggestion is also based on our observation regarding the higher expression of this lncRNA in tumors with lymphatic/ vascular invasion compared with those without lymphatic/ vascular invasion.

We also detected significant correlations between expression levels of *GAS8* and *GAS8-AS1* in both tumor tissues and ANCTs. Such finding is concordant with the recently identified role of *GAS8-AS1* inactivation of *GAS8* expression.  *GAS8-AS1* has a crucial role in keeping the *GAS8* promoter in an active configuration by engaging mixed-lineage leukemia 1 (MLL1)/ WD-40 repeat protein 5 (WDR5) complex (7).

Notably, we reported the diagnostic power of 76% for *GAS8* in gastric cancer which was consistent with the significant down-regulation of this gene in gastric tumor tissues compared to ANCTs. The diagnostic potential of this gene has not previously assessed in human malignancies. However, a previous study evaluated the appropriateness of its antisense RNA in the differentiation of PTC from nodular goiters. Authors have reported that the diagnostic power of plasma levels of this lncRNA was 0.746 in the prediction of lymph node metastasis (6). A future perspective of the current research might be an evaluation of GAS8 transcript levels in the plasma samples of gastric cancer patients to appraise the potential in non-invasive detection of cancer. 

All-told, our data demonstrate dysregulation of *GAS8* in gastric cancer in association with some tumor features and its potential as a marker for diagnosis of gastric cancer. Future functional studies are needed to verify our results.
